# Study of structural stability and damaging effect on membrane for four Aβ42 dimers

**DOI:** 10.1371/journal.pone.0179147

**Published:** 2017-06-08

**Authors:** Wei Feng, Huimin Lei, Jiarui Si, Tao Zhang

**Affiliations:** 1 School of Biomedical Engineering, Tianjin Medical University, Tianjin, China; 2 School of Continuation Education, Tianjin Medical University, Tianjin, China; 3 School of Basic Medicine, Tianjin Medical University, Tianjin, China; University of Akron, UNITED STATES

## Abstract

Increasing evidence shows that Aβ oligomers are key pathogenic molecules in Alzheimer’s disease. Among Aβ oligomers, dimer is the smallest aggregate and toxic unit. Therefore, understanding its structural and dynamic properties is quite useful to prevent the formation and toxicity of the Aβ oligomers. In this study, we performed molecular dynamic simulations on four Aβ42 dimers, 2NCb, CNNC, NCNC and NCCN, within the hydrated DPPC membrane. Four Aβ42 dimers differ in the arrangements of two Aβ42 peptides. This study aims to investigate the impact of aggregation pattern of two Aβ peptides on the structural stability of the Aβ42 dimer and its disruption to the biological membrane. The MD results demonstrate that the NCCN, CNNC and NCNC have the larger structural fluctuation at the N-terminus of Aβ42 peptide, where the β-strand structure converts into the coil structure. The loss of the N-terminal β-strand further impairs the aggregate ability of Aβ42 dimer. In addition, inserting Aβ42 dimer into the membrane can considerably decrease the average APL of DPPC membrane. Moreover this decrease effect is largely dependent on the distance to the location of Aβ42 dimer and its secondary structure forms. Based on the results, the 2NCb is considered as a stable dimeric unit for aggregating the larger Aβ42 oligomer, and has a potent ability to disrupt the membrane.

## Introduction

Alzheimer’s disease (AD) is an irreversible and progressive neurodegenerative disorder that is characterized by the impairment of memory, attention and executive function [1,2]. The cognitive impairment is mainly caused by the formation of senile plaques, which has been considered as a hallmark in the development of AD [3,4].The plaque deposits are primarily composed of insoluble fibrils that are aggregated from various amyloid-β (Aβ) peptides with 39–42 amino acids. Among these Aβ peptides, Aβ42 is an important alloform due to its high propensity for aggregation [5]. However, the increasing evidence indicates that the insoluble fibrillar aggregates are not the toxic species, but smaller soluble Aβ oligomers are the neurotoxic species in AD [6].

Although the precise molecular mechanism how the Aβ oligomers induce neurotoxicity remains unclear [7], some experimental and theoretical studies revealed that the Aβ oligomers can affect membrane integrity and enhance membrane permeability when associated with the neural membrane [8]. This alteration on the membrane permeability can lead to excessive leakage of ions, and the subsequent imbalance of ionic homeostasis results in the dysfunction and death of neurons [9]. Therefore, the full investigation of interaction between the Aβ oligomers and lipid membrane will be crucial for clearly elucidating the mechanism of the Aβ oligomers toxicity and effectively preventing the Aβ-induced neuronal damages.

In addition, the Aβ oligomers with different structural features exhibit different level of cytotoxicity. Therefore, the structural characterization is also essential for understanding the role of the Aβ oligomers in Alzheimer’s disease and inhibiting their toxicity [10]. Many advanced experimental techniques have been applied to determine and describe the structures of Aβ oligomers. Depending on these techniques, the molecular architecture of Aβ oligomers was probed in more detail than before. However, completely determining the structure of Aβ oligomers and elucidating their aggregation process still remain challenging because of fast aggregation, flexibility and heterogeneous ensemble of the Aβ oligomers [11]. As a complement to the experimental methods, computer simulation methods can provide the detailed information on the structural features of the Aβ oligomers at the atomic level [12]. They are also applied to explore the aggregated forms of the Aβ oligomers embedded within the lipid membrane [13] and investigate their effects on the membrane [14]. Combining the experimental and computational results, many structures of Aβ oligomers within the lipid bilayer have been identified. For example, the channel-like structures composed of Aβ tetramers or hexamers have been identified using atomic force spectroscopy and molecular dynamics simulation. This Aβ channel structure can penetrate the membrane and form a trans-membrane pore [15–17]

As the smallest oligomeric and toxic species, Aβ dimer is attracting more and more attentions [18]. Many computational studies were performed to identify the unique structural characteristics of Aβ dimer from other Aβ aggregated forms [19] and investigate the aggregate formation of the Aβ dimer within both the membrane and water [20,21]. These studies revealed that the Aβ peptides in the dimer have a high propensity for β-strand at some specific regions [22]. Moreover, the interpeptide interactions occurred at these regions can act as an important driving force for the dimerization of the Aβ peptides [23]. It implies that the β-strand structure may be necessary for the Aβ dimer formation, which is in good consistent with the experimental observations [24]. Although these studies provided deeper insights into the structure and aggregation of Aβ dimer, it is known a little about the possible structures of the Aβ dimer within the membrane and the interaction between the Aβ dimer and the membrane.

In a previous study that aimed to identify the structures of the Aβ42 oligomers using a global optimization approach with an implicit membrane model, the Aβ42 dimer is found to have four stable aggregated forms [13]. They were denoted as 2NCb, CNNC, NCNC and NCCN according to the arrangements of two peptides within the dimer. The letter N and C represent N- and C-terminus of each Aβ peptide, respectively. In the present study, we use the four structures identified by Strodel *et al*. as starting structures of four Aβ42 dimers to observe their structural stability and aggregation tendency within the membrane environment, as well as their effect on the membrane by using the molecular dynamics simulation with an explicit membrane model. Comparing their structural features and membrane disrupting effect of the four Aβ42 dimers, we investigate the influence of relative position of two Aβ42 peptides on Aβ42 dimerization and the induced membrane disruption. Therefore, the results may be of significant use for better understanding the aggregation process of Aβ oligomers and the membrane disrupting mechanism of Aβ oligomers with different size and shapes. Furthermore, our results may be useful for preventing the formation of Aβ oligomers and inhibiting their toxicity.

## Materials and methods

### Starting structures of four Aβ dimers

The amino acid sequence of Aβ42 is DAEFRHDSGYEVHHQKLVFFAEDVGSNKGAIIGLMVGGVVIA. Based on the distribution of hydrophobic and charged amino acids, the sequence of Aβ42 can be divided into two different parts. The first segment ranges from D1 to K16, corresponding to N-terminus of Aβ peptide. In this region the residues are primarily hydrophilic or charged amino acid. Hence this part can be referred to as the polar region of Aβ42. On the contrary, the second one is from L17 to A42 that mainly consisted of hydrophobic residues and a few polar residues. This segment is called as the C-terminal apolar region that contains the central hydrophobic core and the C-terminus of Aβ42 peptide.

Four stable aggregated forms of Aβ42 dimer identified from the previous study are used as starting structures for molecular simulation. They are denoted as 2NCb, CNNC, NCNC and NCCN, respectively. Although two Aβ peptides aggregate with different arrangements in the dimer, each Aβ peptide adopts the same structure. At the N-terminal polar region there is an antiparallel β-hairpin structure, and the C-terminal apolar region forms a more complex β-sheet structure consisting of three antiparallel β strands. In addition, a short coil segment ranging from Q15 to V18 locates between two β-sheet structures.

**Fig 1:**
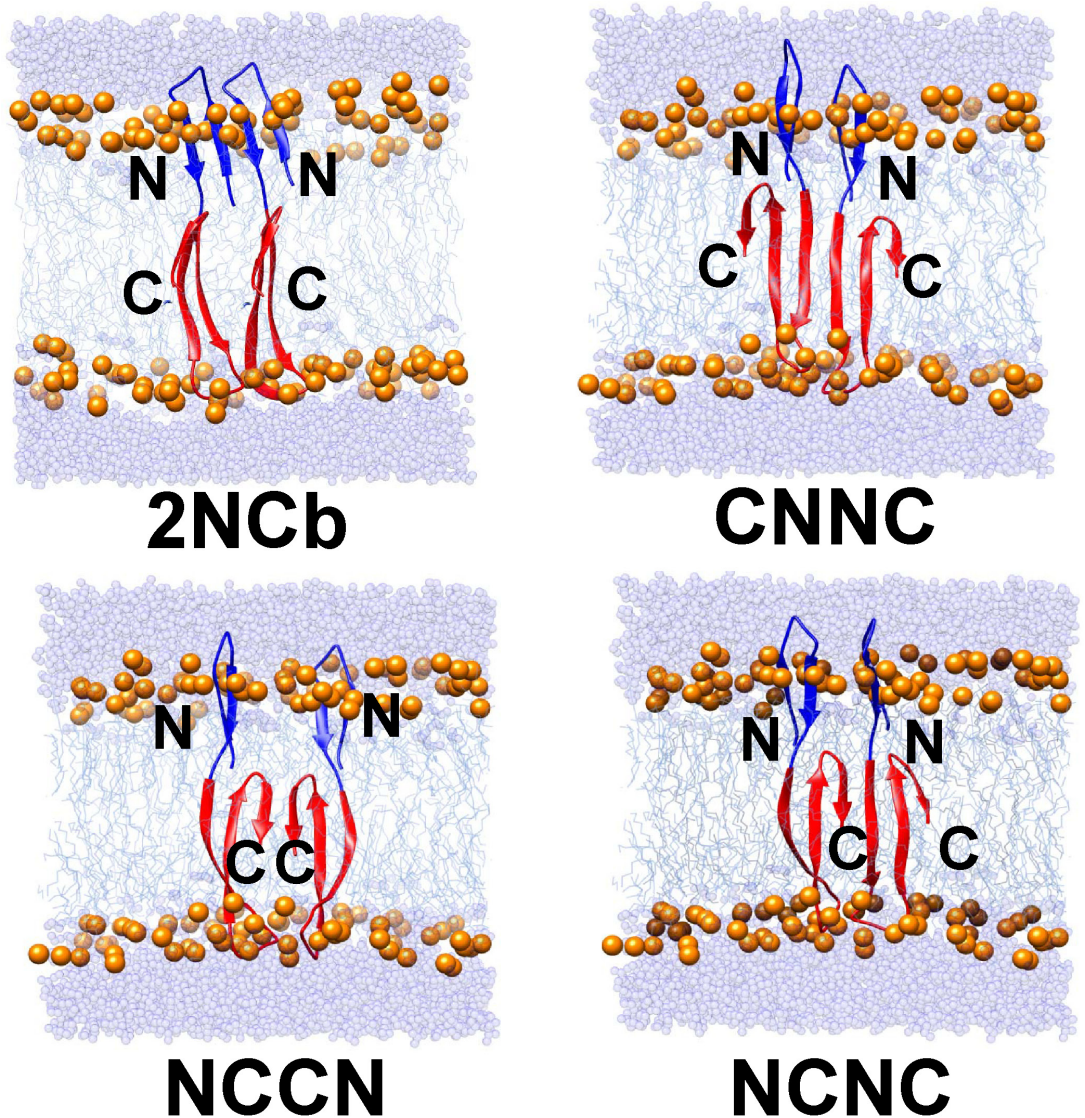
Initial structures of four Aβ42 dimers within the hydrated DPPC membrane. The Aβ42 peptide is shown in carton. The polar and apolar regions of Aβ42 peptide are colored in blue and red, respectively. The water molecules and phosphorus atoms are shown as Van der Waals spheres of different colors and sizes. The water molecules on the surface of DPPC bilayer are colored in light blue, and the phosphorus atoms within the upper and lower layers are colored in orange. The hydrophobic core (acyl chains) in DPPC is displayed as line in cyan.

The starting structures of four Aβ42 dimers were inserted into the hydrated DPPC membrane, respectively. The insert orientation is along the Z-direction perpendicular to the membrane surface. The inserted depth of each Aβ42 dimer is consistent with the previous study ^[^^13^^]^. The N-terminal polar region of the Aβ42 peptide is located within the lipid head region of the DPPC membrane and the orientation of two β-strands is vertical to the phosphorus atoms on the upper layer. The C-terminal apolar region is embedded into the hydrophobic core of DPPC. Because three consecutive polar residues are present in a loop structure (G25-A30), this loop structure is positioned within the head group region on the lower layer. Fig 1 shows the starting structure of four Aβ42 dimers and their location within the hydrated DPPC membrane.

In this study, we only select the pure DPPC membrane as the lipid bilayers to investigate the structures of Aβ42 dimers within the biological membrane. There are mainly two reasons for this selection. Firstly, due to that the DPPC molecule is a zwitterionic phospholipid, it can’t affect local pH and induce protonation state changes on the Aβ peptide. Secondly, a study investigating the interaction between Aβ42 peptide and two different phospholipid bilayers has revealed that the addition of anionic bilayers can lead to a significant change in the secondary structure of the peptide, whereas the addition of zwitterionic membrane does not affect peptide structure [25].

### Molecular dynamics simulation

All MD simulations of Aβ42 dimers were performed by Gromacs software (version of 5.0-rc1) [26] using GROMOS96 53A6 force filed [27] and the modified Berger force field parameters for lipid molecules [28]. A snapshot of a lipid bilayer containing 128 DPPC molecules after 40 ns of simulation was taken as the initial structure of lipid membrane [29]. The Aβ42 dimer was firstly inserted into the DPPC membrane according to the protocol applied in the membrane protein simulations [28]. Then the whole system composed of DPPC lipids and Aβ42 dimer was solvated with SPC water molecules, and the water molecules located in the interior of the membrane were removed from the DPPC membrane. Finally, the Na+ and Cl- ions as the counterion were added to neutralize the total charge of system and mimic the physiological salt concentration of 0.1 M NaCl solution.

Prior to the simulation, each system was equilibrated by a two-step protocol. A NVT equilibration was firstly performed at 323K for 1000 ps using the v-rescale thermostat method with a coupling constant of 0.1 ps. Subsequently, the system was equilibrated under NPT conditions for 50 ns, during which the Nose-Hoover thermostat was used to control the temperature of the whole system. The semi-isotropic Parrinello-Rahman method was used to maintain a constant pressure of 1 bar in z-direction and x-y plane with a time constant of 5.0 ps. Throughout the equilibration process, the Aβ42 dimer and phosphorous atom of each DPPC molecule were restrained with a force constant of 1000 kJ mol^-1^ nm^-2^. Following NVT and NPT equilibration, the MD simulations for each Aβ42 dimer embedded in the hydrated DPCC membrane was performed for 400 ns, during which all restraints imposed on the Aβ42 peptides and phosphorous atoms were removed. In order to clearly explore the impact of Aβ dimer on the membrane, a simulation of the hydrated DPPC bilayer was performed for 400 ns under the same conditions. In all simulations, the long-range electrostatic interaction was calculated using PME method under periodic boundary conditions.

### Analysis

The structural stability of Aβ42 dimer in the DPPC bilayer was analyzed in terms of its location within the lipid bilayer and secondary structure changes during the simulation. DSSP method [30] was applied to define the secondary structure of Aβ42 peptide. Additionally, 40,000 structures were extracted from the 400 ns MD trajectory for performing cluster analysis. The cluster analysis was done using Gromos algorithm [31], by which the structures with a Cα RMSD less than 2.5 Å were classified into the same cluster.

In addition, the properties characterizing the structural and dynamic nature of membrane were analyzed to explore the changes induced by the Aβ42 dimer. The area per lipid was calculated using GRIDMAT-MD package, a grid-based membrane analysis tool [32]. The deuterium order parameter S_CD_ was also computed for the lipid acyl chains of DPPC. The residence time of DPPC molecules around the Aβ42 dimers was also calculated to describe the fluidity of the membrane.

## Results

The simulation results show that the four Aβ42 dimers remain inside the membrane throughout MD simulation. But due to different aggregation patterns of two Aβ42 peptides, four Aβ42 dimers displayed different degree of structural stability and effects on the lipid membrane.

### Movement of Aβ42 dimers in DPPC membrane

**Fig 2:**
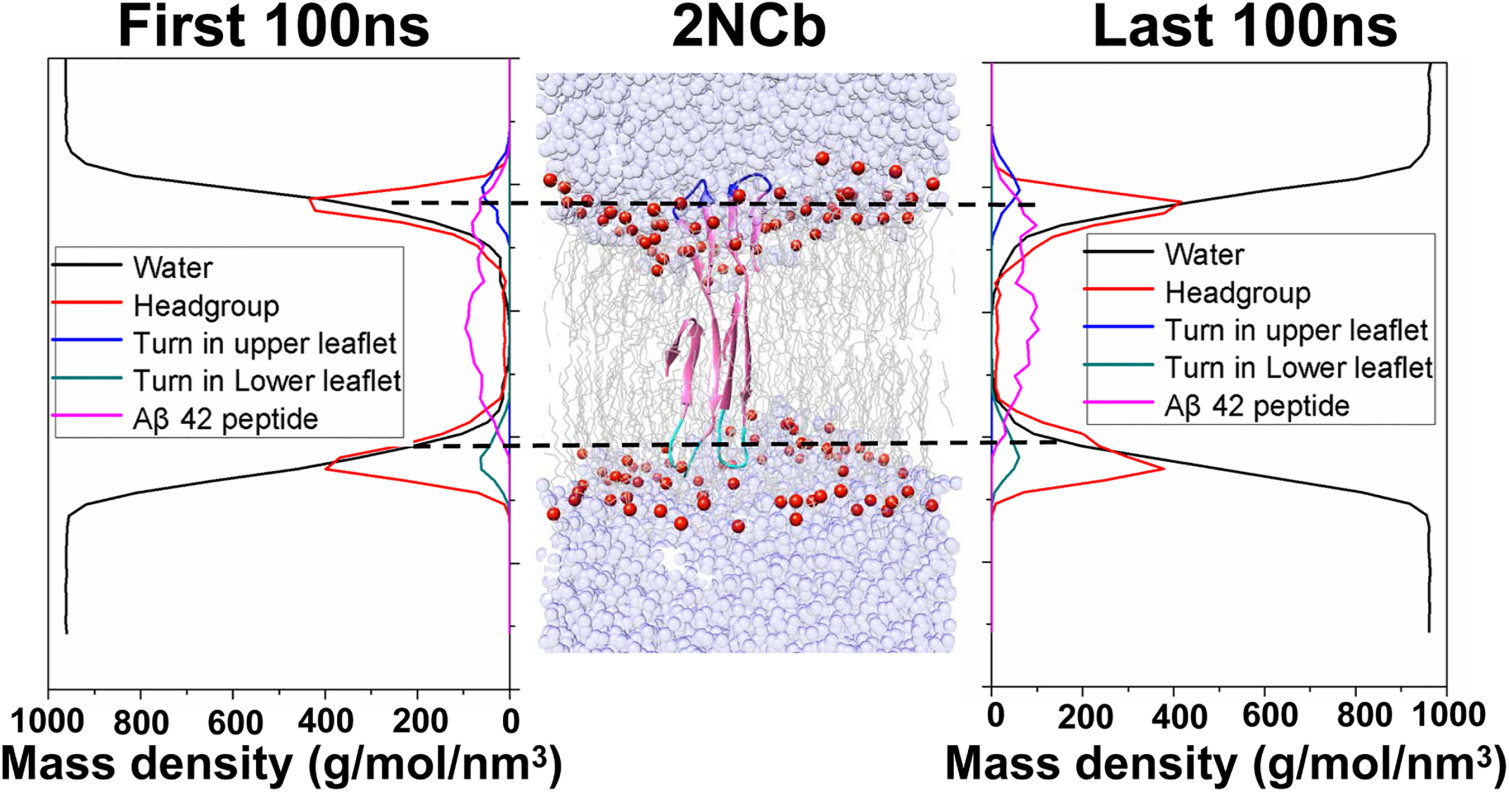
Comparison of the mass density profiles for the 2NCb, water (black) and head-group of DPPC (red) during the fist and last 100 ns of simulation. According to the position of the peptide within the DPPC membrane, the Aβ42 peptides are divided into three sections, two turns located in the upper and lower leaflet of the DPPC membrane and the remaining section of Aβ42 peptide. A snapshot from the trajectory is used to illustrate the exact position of three sections within the DPPC. The turns in the upper and lower leaflets are colored in blue and cyan, respectively, the remaining section in pink.

The mobility of four Aβ42 dimers within the lipid membrane is firstly investigated by comparing mass density profiles of the Aβ dimer, water molecules and lipid head group atoms. We take the 2NCb as an example to describe how to define the movement of Aβ42 dimer from the analysis of density profile. Fig 2 shows the comparison of the mass density profiles between the 2NCb dimer, the water and the lipid head group atoms, along with a structure extracted from the MD trajectory. All density profiles are calculated from the first and last 100 ns of the trajectory, respectively.

Based on the comparison, we find that both at the beginning and end of the simulation the density profile curve of Aβ42 peptide are mainly located between the density profiles of head group atoms in the two leaflets of the membrane. It implies the 2NCb did not move dramatically along the z-direction of the DPPC membrane during the simulation. In addition, we observe that the density profiles of two turn structures on the N- and C-terminus almost completely overlap with the density profiles of the head group atoms in the upper and lower leaflets, indicating that these two turn structures are inserted into the lipid head group regions as illustrated by the structure presented in the Fig 2. Because in the two turn regions most residues are the hydrophilic and polar amino acids, their side chains may interact with the lipid head group atoms and water molecules. These interactions enable the 2NCb dimer to be properly and stably embedded into the lipid bilayer. The comparison of density profiles for other three Aβ dimers is displayed in S1 Fig. Similar to the 2NCb dimer, other three Aβ dimers are also embedded well into the membrane. This result is agreement with the previous study that Aβ42 dimer is favored within the membrane [33].

Based on the analysis above, we can conclude that although the four Aβ42 dimers aggregate with different patterns of Aβ peptides, they can stably stay in the membrane. The final snapshots of four simulations are shown in S2 Fig. As shown by this figure, the two loops at the N- and C- terminus are mainly located within the lipid head group region in the upper and lower leaflets, which is consistent with the binding model of Aβ peptide and lipid in a solid-state NMR study [34]. Since these two loop regions are composed of more charged and polar amino acids, these residues have a strong tendency to stay in the polar head-group regions [35]. Via these interactions, the Aβ42 peptide can be stably anchored into the lipid head group regions. These results indicate that the aggregated arrangement of Aβ peptides does not result in the exit of Aβ peptide from the membrane bilayer. However, S2 Fig also shows that the β-sheet structure at the N-terminal region has partially unfolded for three Aβ42 dimers, CNNC, NCCN and NCNC. It means that the structural change may occur on these Aβ42 dimers.

### Structural stability of Aβ42 dimers within the membrane

To further investigate the structural stability of the Aβ42 dimers in the environment of lipid bilayer, we compare their structural fluctuation during the simulation. S3 Fig shows the comparison of root-mean-square deviation (RMSD) values of the backbone atoms among the four Aβ42 dimers. From this figure we can observe that four Aβ42 dimers undergo different degree of the structural fluctuation. Thus the different aggregation patterns of Aβ peptides may have a potential effect on the structural stability of Aβ42 dimmer.

Subsequently, we calculate root-mean-square fluctuations (RMSF) of the backbone atoms of four Aβ dimers and plot the RMSF values against each residue in S4 Fig. Although the residues with large RMSF values are almost identical for the four Aβ42 dimmers, the maximum is different from each other. The residues on the NCCN and NCNC exhibit the larger RMSF values compared with ones on the CNNC and 2NCb. As shown by these results, the four Aβ42 dimers exhibit different extent of structural flexibility within the membrane. The NCCN and NCNC have the more flexible regions, in which the local structure changes may occur.

### Secondary structure alteration of Aβ42 dimers

**Fig 3:**
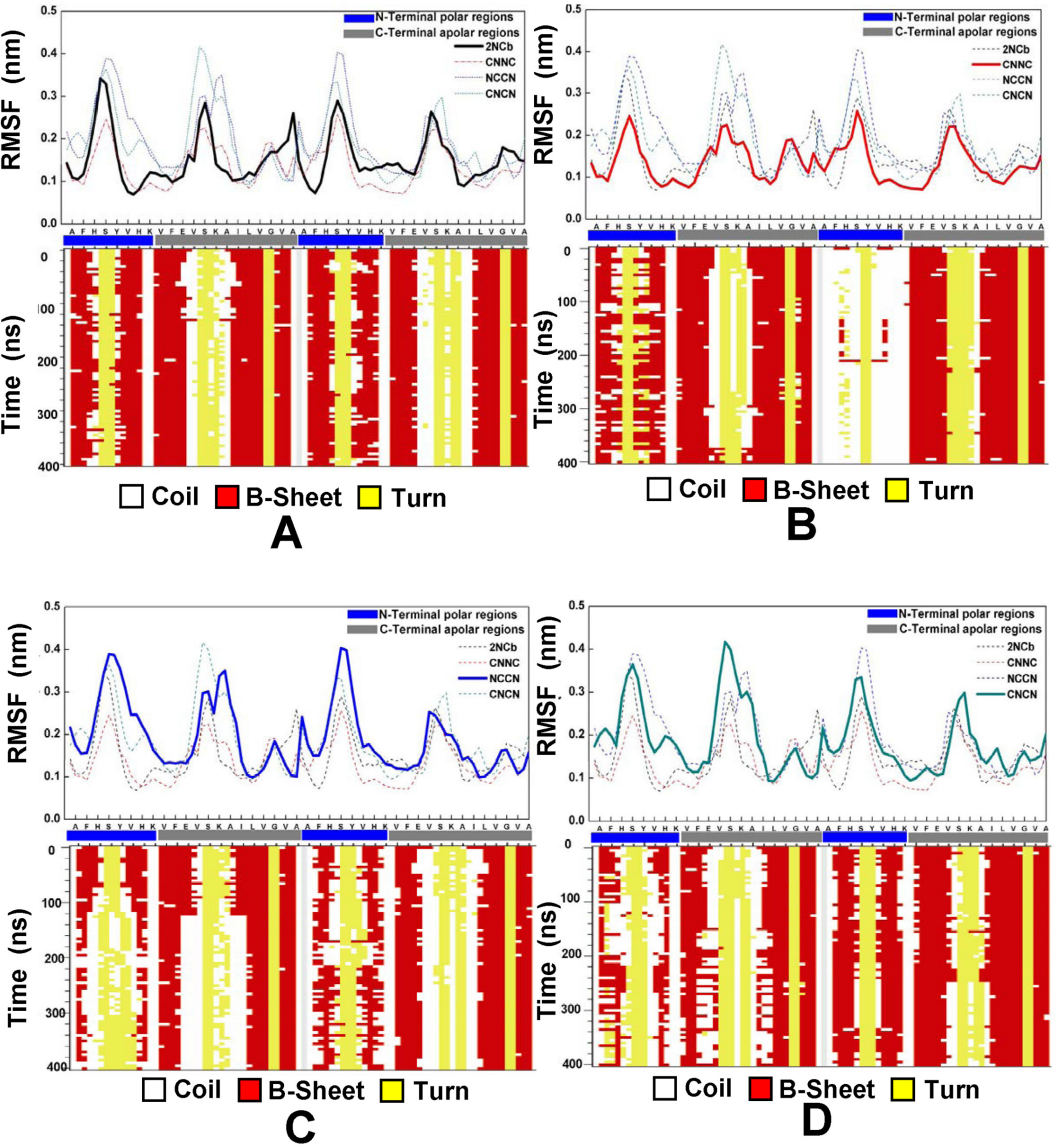
**Secondary structure evolution of the four Aβ42 dimers, 2NCb (A), CNNC (B), NCCN (C) and NCNC (D), along with their respective RMSF**. In the plot of RMSF, X-axis represents the residue number. The residues located in the N-terminal polar region of the Aβ42 peptide are marked with a blue bar, and the residues in the C-terminal apolar region are marked with a gray bar.

We further investigate the change of secondary structure occurring in the flexible regions. Here we consider three common types of secondary structure, β-strand, turn and coil. The secondary structure evolution of the four Aβ42 dimers is shown in Fig 3, together with their plots of RMSF. As described in the section above, the Aβ42 peptide can be divided into two segments, the N-terminal polar and the C-terminal apolar region. This figure shows that, at the C-terminal apolar region, the major structural changes are the reciprocal conversion between turn to coil and from β-strand to coil for the NCCN and NCNC dimers. Additionally, the structural change from β-strand to coil is also frequently observed at the N-terminal region. Although the secondary structure evolution is similar among the four Aβ42 dimers, the extent of structural change is more different from each other. As demonstrated in the analysis of RMSD that the 2NCb undergoes less structural fluctuation, Fig 3 shows both at the N-terminal and C-terminal regions the 2NCb has very little structural change.

This observation can also be validated by comparing average content of secondary structures (S5 Fig). The 2NCb dimer has the highest content of β-strand (52%) comparing with other three Aβ42 dimers. On the contrary, the coil contents are 36%, 30% and 28% for the NCCN, CNNC and NCNC, respectively, which are higher than one for the 2NCb (26%). In addition, the content of turn structure is similar varying from 21% to 23% for the four dimers. These comparisons imply that the conversion from β-strand into coil structure takes place more in the NCCN, CNNC and NCNC leading to the corresponding increase in the coil content and the decrease of β-strand, whereas such structural change is little found in the 2NCb.

Based on the analysis, we identified that the structural change from β-strand to coil and the reciprocal conversion between coil and turn are two major types of structural change occurring in the four Aβ42 dimers. But the conversion from β-strand into coil occurs more in the three Aβ42 dimers, namely the NCCN, CNNC and NCNC. Therefore, we conclude that the larger structural fluctuations in these three Aβ42 dimers are caused by the structural change from β-strand into coil, especially at the N-terminus of the NCCN and NCNC, where the β-strand structure partially shifts into the coil structure.

### Clustering analysis of Aβ42 dimer structures

We performed the cluster analysis to further describe the structural change of Aβ42 dimer. The total 40,000 structures were extracted from the MD trajectory and were mutually compared to classify them according to the structural similarity. Using a cutoff of 0.25nm, the structures of four Aβ42 dimers are respectively divided into 4, 10, 9 and 7 clusters for the 2NCb, CNNC, NCCN and NCNC. The clustering results are summarized and listed in Table 1. The results show a large homogeneity for the 2NCb structures that about 94.3% is classified into the first cluster and only 5.7% structures are from other clusters. On the contrary, other three Aβ42 dimers illustrate a structural heterogeneity that most of structures are from several clusters. The clustering results also validate the above analysis of structural stability.

**Table 1:** The clustering results of four Aβ42 dimmers structures extracted from the MD trajectory including total cluster numbers, the number and corresponding percentage of structure in each cluster.

	2NCb	CNNC	NCCN	NCNC
Total Cluster Number	4	0	9	7
**Cluster 1**	37730 (94.3%)	35570(88.9%)	28965 (72.4%)	31487 (78.7%)
**Cluster 2**	2148 (5.4%)	2656 (6.6%)	7148 (17.8%)	7421 (18.6%)
**Cluster 3**	72 (0.2%)	702 (1.8%)	2691 (6.7%)	885 (2.2%)
**Cluster 4**	50 (0.1%)	483 (1.2%)	977 (2.4%)	201 (0.5%)
**Cluster 5**		300 (0.7%)	105 (0.2%)	4 (0.01%)
**Cluster 6**		165 (0.4%)	58 (0.1%)	2 (0.005%)
**Cluster 7**		84 (0.2%)	47 (0.1%)	1 (0.005%)
**Cluster 8**		33 (0.1%)	7 (0.02%)	
**Cluster 9**		4 (0.01%)	4 (0.01%)	
**Cluster10**		3 (0.01%)		

**Fig 4:**
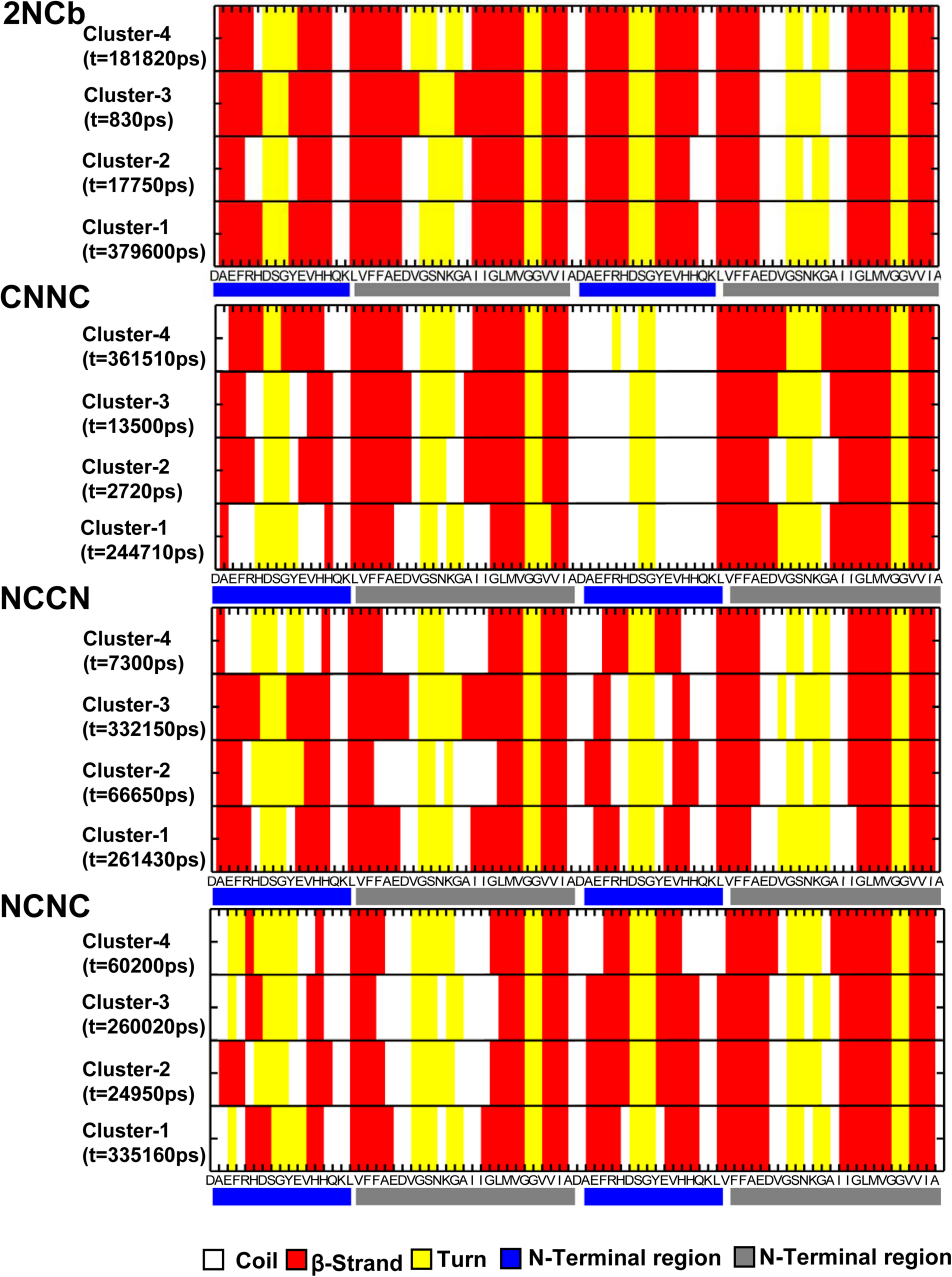
The secondary structure of central structure of top 4 clusters for four Aβ42 dimers. X-axis represents the residue number. The residues located in the N-terminal polar region of the Aβ42 peptide are marked with a blue bar, and the residues in the C-terminal apolar region are marked with a gray bar.

To explore the structural difference between several major clusters, we extract the central structure of clusters to compare their secondary structures. Fig 4 displays the secondary structure of central structure from top 4 clusters against each residue. We can clearly find that the major secondary structure at the N-terminal region is β-strand in the central structures of 2NCb. However, for the CNCN, NCCN and NCNC, the β-sheet structure at the N-terminal region is changed to some extent into coil or turn structures. This result is also consistent with the secondary structure evolution of the four Aβ42 dimers. Moreover, we find that the conversion of β-strand structure is primarily located in the border of β-strand rather than the center. The research on the role of specific residues in the conversion of β-strand using umbrella sampling method is currently underway in our laboratory.

### Distribution of secondary structures in Aβ42 peptides

**Fig 5:**
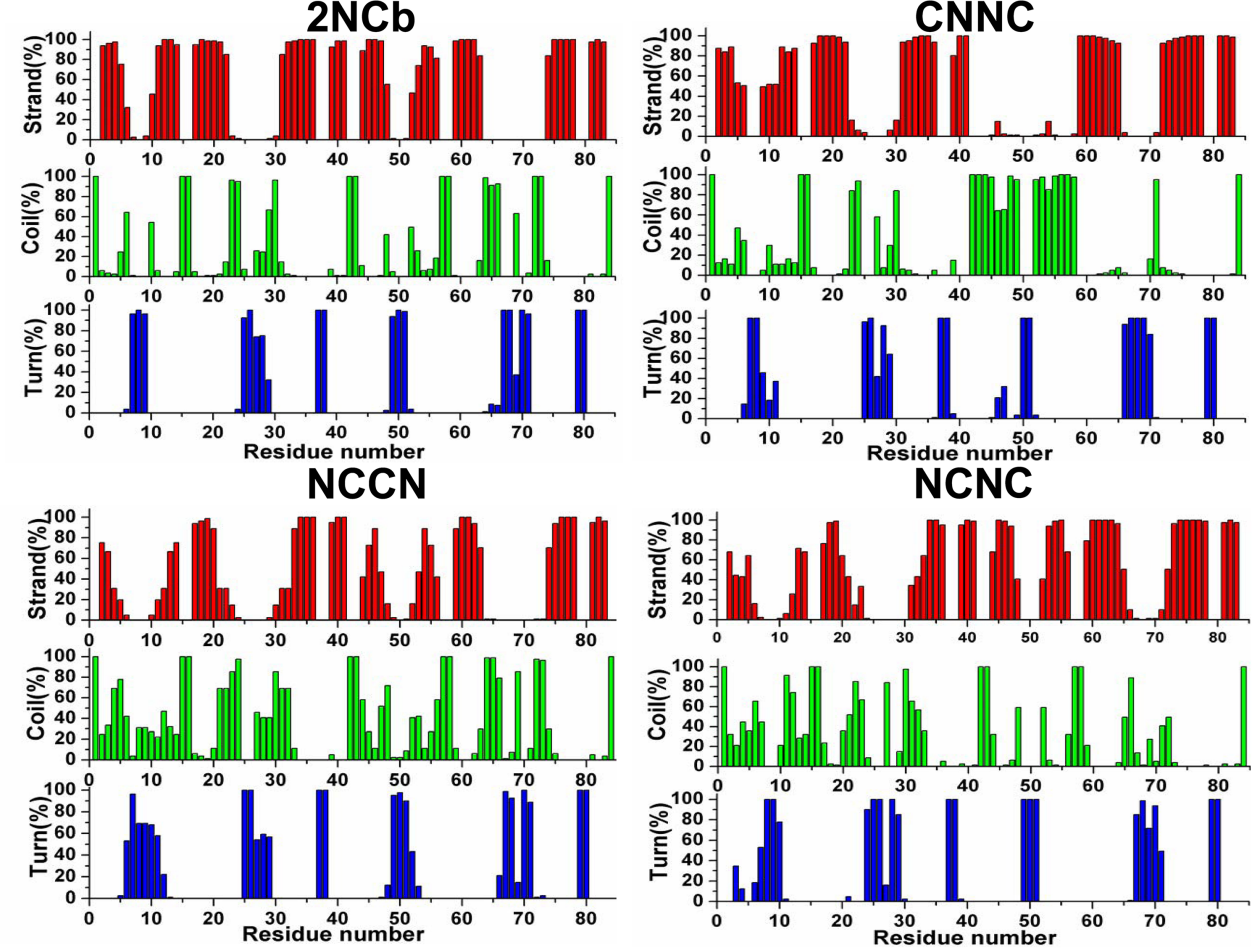
Comparison of structural propensity for β-strand (red), Coil (green) and Turn (blue) among the four Aβ42 dimers.

Then we further performed the secondary structures propensity analysis for each residue on the Aβ42 peptides. Fig 5 shows the secondary structures propensity plots of the four Aβ42 dimmers. From the propensity distribution for the 2NCb, we identify five regions with high propensity for β-strand in the Aβ42 peptide, two regions (residues 2–5 and 11–14) positioning at the N-terminus polar segment and three regions (residues 17–22, 31–36 and 39–41) being located at the C-terminus apolar part. However, when comparing the propensities of β-strand and coil for the same residue, we find that, in the case of the NCCN, CNNC and NCNC, the residues in the two regions at the N-terminus of the Aβ42 peptide display a decrease in the β-strand propensity and a corresponding increase in the coil propensity. On the contrary, the residues in the three regions at the C-terminus have the similar β-strand and coil propensities for the four Aβ42 dimers.

The above results indicate that the β-strand structure at the N-terminus of the Aβ42 peptide may have different stability when two Aβ42 peptides aggregate in different patterns. For the 2NCb the β-strand structure at the N-terminus presents almost throughout the simulation, whereas in the case of the other three Aβ42 dimers the β-strand in this region converts into the coil. Because the four Aβ dimers differ only in the aggregation pattern of two Aβ42 peptides, it may be the reason for the different stability of the β-strand structure at the N-terminus of the Aβ42 peptide.

### Analysis of hydrogen bond interaction

The results above show that the β-strand structures at the N-terminus have different stability properties depending on the different aggregate patterns of the Aβ42 peptides. Because hydrogen bond interaction plays the primary role in maintaining the β-strand structure, we explore the reasons for the instability of the β-strand structure by analyzing inter- and intra-peptide hydrogen bonds within the Aβ42 dimer. As demonstrated above, there are five β-strand structures in the Aβ42 peptide, i.e. two β-strands (β-strand 1 and 2) at the N-terminal end and three β-strands (β-strand 3–5) at the C-terminal part. Therefore, when analyzing intra-peptide hydrogen bonds, we separately calculate the hydrogen bond interactions between two β-strands at the N-terminus and among three β-strands at the C-terminus, whereas for the analysis of inter-peptide hydrogen bonds we only investigate the hydrogen bond interactions between the same β-strands at the two Aβ42 peptides.

Table 2 lists the total number of hydrogen bonds formed during 400 ns simulation and the related information on the β-strands involving the formation of hydrogen bonds. Regarding the intra-peptide hydrogen bonds there is an insignificant difference for the four Aβ42 dimers, suggesting that the different stability of β-strands at the N-terminal part could not be related with the intra-peptide interactions between the adjacent β-strands at the same Aβ42 peptide. However, as for the inter-peptide hydrogen bonds, there is a distinct difference in the position of the hydrogen bonds among the four Aβ42 dimers. For the 2NCb, the inter-peptide hydrogen bonds are only formed between the β-strands 1–2 of two Aβ42 peptides, whereas the inter-peptide hydrogen bonds are only found between the two β-strand 3 regions, the two β-strand 5 regions and the β-strand 3 and β-strand 5 regions for the CNNC, NCCN and NCNC, respectively. Therefore, the absence of inter-peptide hydrogen bonds at the N-terminus may result in the disruption of β-strands at this region for the three Aβ42 dimers.

**Table 2:** Total number of hydrogen bonds including intra- and inter-peptide hydrogen bonds formed during 400 ns simulation and the related information on the β-strands involving the formation of hydrogen bonds.

	2NCB	CNNC	NCCN	NCNC
**Intra-Aβ42 peptide-A**				
**β-strands 1–2**	6.37±1.43	6.71±1.44	4.37±1.99	6.02±1.59
**β-strands 3–4**	5.32±0.73	5.50±0.70	3.80±1.36	3.63±0.92
**β-strands 4–5**	1.96±0.36	2.03±0.43	1.88±0.42	1.96±0.36
**Intra-Aβ42 peptide-B**				
**β-strands 1–2**	5.70±1.39	5.43±1.47	5.53±1.74	6.08±1.45
**β-strands 3–4**	5.42±0.68	5.36±0.76	4.90±0.86	5.56±6.54
**β-strands 4–5**	1.91±0.38	1.90±0.43	1.92±0.40	1.91±0.38
**Inter-Aβ42 peptide-AB**				
**β-strands 1–2**	5.21±1.47	0	0	0
**β-strand 3**	0	4.55±0.71	0	0
**β-strand 4**	0	0	0	0
**β-strand 5**	0	0	2.65±0.87	0
**β-strand 3–5**	0	0	0	3.81±0.88

In summary, the results show that the four Aβ42 dimers exhibit different structural stability within the lipid membrane. The NCCN, CNNC and NCNC have the larger structural fluctuation particularly at the N-terminus, where the β-strand structure converts into the coil structure. Furthermore, this structural change is directly caused by the absence of inter-peptide hydrogen bonds at this region. The previous studies revealed that the formation of β-strand structure at the N-terminus [36] and the interactions between the N-terminus [37] play the vital role in the aggregation of Aβ42 peptides. Therefore, when the β-strand structures at the N-terminal part of Aβ42 peptide disappear due to no formation of the inter-peptide hydrogen bonds, the Aβ42 dimer may not be able to further aggregate into stable oligomeric structure. According to this assumption, the 2NCb may be an important conformation of Aβ42 dimer for the formation of higher oligomers, which is agreement with the results from the theoretical study [13] and the fibril structures obtained using cryo-electron microscopy [38].

### Effect of Aβ42 dimers on membrane structure

The simulation results reveal that the four Aβ42 dimers undergo the different extents of structural fluctuation and the loop structures connecting two β-strands have the higher flexibility than other regions. It implies that the high flexibility of the loop structures may have a significant effect on the integrity of membrane, particularly in the head-group regions due to that two loop structures at the N- and C-terminus are mostly buried into the phospholipid head-group regions. To probe the ability of the Aβ42 dimers to disrupt the membrane bilayers, we calculate the value of area per lipid (APL) of DPPC membrane that contains the embedded Aβ dimer and compare with that of pure DPPC membrane. When comparing the APL, we split the membrane into three continuous regions, the core region that is within the distance of 5Å from the embedded Aβ42 peptide, the intermediate region that is positioned within the distance between 5 and 10 Å as well as the remote region that is located with the distance more than 10 Å.

**Fig 6:**
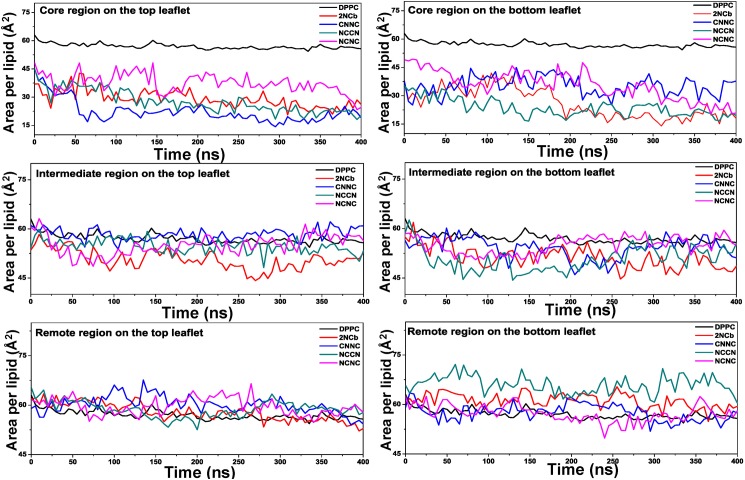
Comparison of average APL for the three regions in DPPC membranes containing the four Aβ42 dimers respectively with the pure DPPC membrane. Three regions are considered, core, intermediate and remote region, on the top and bottom leaflets of DPPC membrane.

The comparison results of the average APL between the DPPC membranes containing different Aβ42 dimers and the pure DPPC membrane are shown in Fig 6, from which we find that the extent of change in the average APL is also different within the three regions.

Within the core region, the average APL values of both upper and lower leaflets are greatly decreased in the DPPC membranes embedded with the Aβ dimer. It indicates that the insertion of Aβ peptide into the membrane has a destroying effect on the DPPC membrane nearby the Aβ42 dimer. However, around the intermediate region and the remote region, the DPPC membranes containing the Aβ dimer have the similar average APL value to the pure membrane. It implies that the destroying effect caused by the embedment of Aβ dimer is only limited within the neighborhood of the Aβ peptide, and probably related with the two loop structures located at the head-group regions.

**Fig 7:**
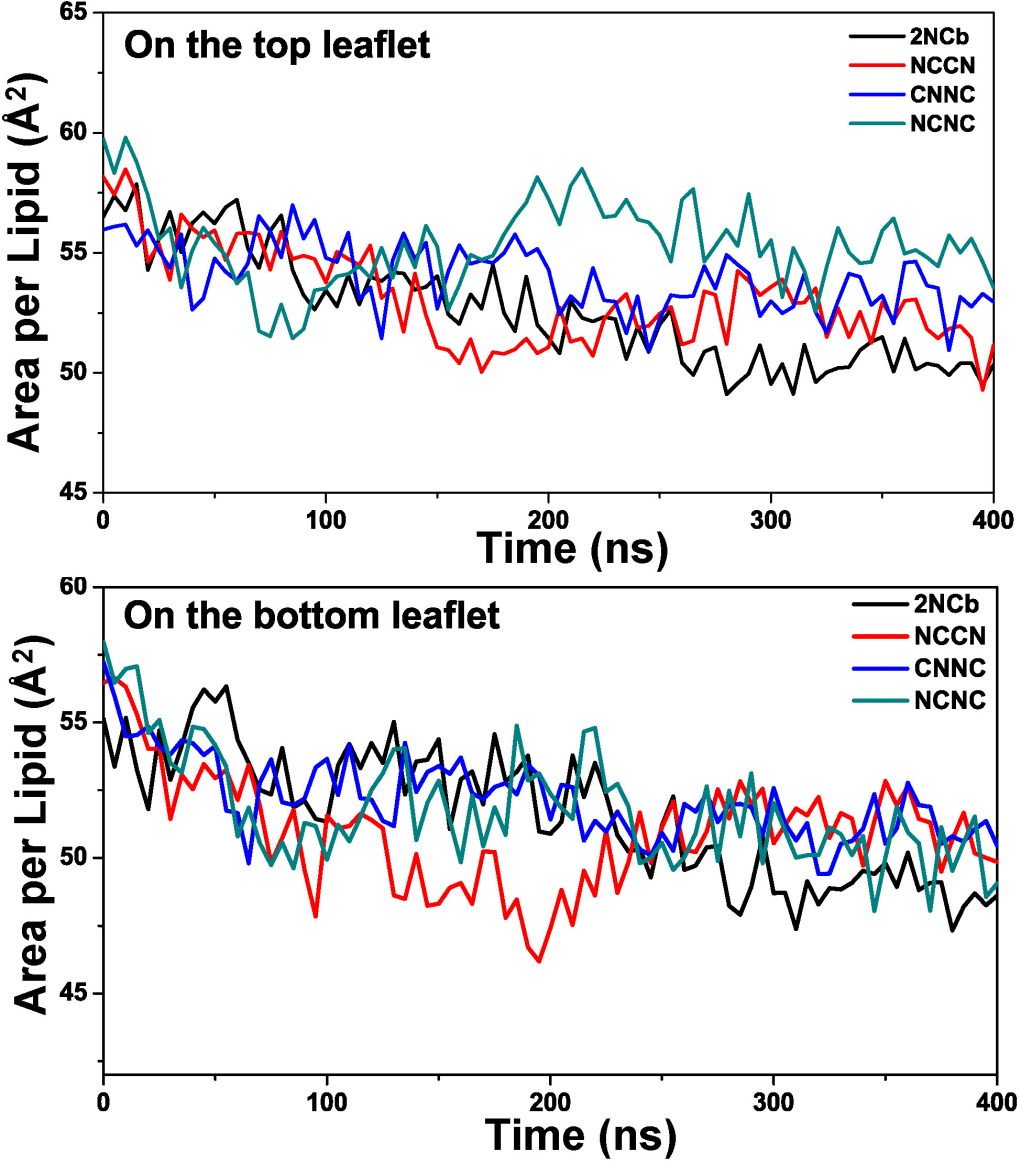
Comparison of average APL on the top and bottom leafets for four DPPC membranes containing the four Aβ42 dimers, respectively.

Additionally, the evolution of secondary structure of the Aβ42 peptide displays that the β-strands at the N-terminal regions have different extent of structural instability. This instability may be capable of disrupting membrane organization. In order to describe this effect, we further compare the average APL value of DPPC membranes, into which the four Aβ dimers with different aggregated arrangements of Aβ peptides were respectively inserted. The comparisons of the average APL for the top and bottom leaflets are shown in Fig 7.

For the top leaflet, we observe that the membranes inserted with the 2NCb and NCCN have the smaller APL value comparing with CNNC and NCNC. But the same trend is not observed on the bottom leaflet of the membrane, where the C-terminus of the Aβ42 peptides is present. To further validate this observation, we extended the MD simulation for 150 ns. From the extending trajectory, the APL on the top and bottom leaflets was calculated at the interval of 500 ps. The comparison of APL between the DPPC membranes inserted with four Aβ42 dimers is shown in S6 Fig. There is a similar trend as observed in the 400 ns MD simulation and the APL value is significantly decreased on the top leaflet of DPPC membrane inserted with the 2NCb and NCCN. But on the bottom leaflet, except for the CNNC, other three Aβ42 dimers have the comparable APL values. Based on the secondary structure analysis, we can find that for the 2NCb and NCCN the residues at the N-terminal region of two Aβ42 peptides have simultaneously high potential to form β-strand structure. The ratio of propensity of β-strand between two Aβ42 peptides within a dimer is 1.0 and 1.3 for the 2NCb and NCCN, which are smaller than one of the CNNC and NCNC (27.5 and 2.0). Therefore, it indicates that this discrepancy of APL reduction may be related to the formation of β-strand structures at the N-terminus of the Aβ42 peptides. Comparing with the β-strand structure that is the rigid structural form, the more disordered structures may have a less effect on the membrane and consequently lead to a little decrease of the average APL.

Furthermore, we measured deuterium order parameter across lipid acyl chain. The order parameter reflects the orientation and ordering of the lipid tails in the bilayer with respect to the bilayer normal. S7 Fig shows the average S_CD_ as function of the carbon atom along the lipid tails for the membranes inserted with four Aβ42 dimers and pure DPPC membrane. Previous experimental results are also shown in S7 Fig for comparison, including NMR measurements of pure DPPC sn-2 chain at 323K [39] and deuterium magnetic resonance of DPPC with spin-labels [40]. From the S7 Fig, we can find that for both the sn-1 and sn-2 chains the value of deuterium order parameter has a similar trend in the DPPC membrane inserted with Aβ42 dimer and pure DPPC membrane. Although the value of deuterium order parameter of DPPC inserted with 2NCb is slightly higher than one with other Aβ42 dimers, this value at the sn-2 chain is within the range between two experimental values. The results imply that the insertion of Aβ42 dimer into the DPPC membrane did not cause significant alteration on the order of membrane.

### Effect on membrane fluidity

Besides analyzing the structural change of lipid membrane, we further investigate the change of membrane fluidity by calculating residence time of DPPC molecules. In this study, the calculation of residence time of DPPC molecules is based on the frequency of DPPC molecules within a defined range. We firstly plot radial distribution functions (RDF) for DPPC molecules with respect to Aβ42 dimer. Because the Aβ42 peptide is inserted into the center of DPPC lipid membrane, RDF curve of DPPC molecules around Aβ42 dimer appears several peaks within the range between 0.5 ns and 3.0 ns as shown in S8 Fig. Then the distance corresponding to 80% of area under the RDF curve was used to select the DPPC molecules, whose frequency is used to calculate the residence time of DPPC membrane. The residence time of DPPC membranes inserted with different Aβ42 dimer is 457.3 ps for the 2NCb, 130 ps for the CNNC, 131.8 ps for the NCCN and 412.1 ps for the NCNC. We can find that the residence time of DPPC membrane inserted with the 2NCb is highest among four Aβ42 dimers. The high residence time reflects the reduced lipid fluidity, which is a typical indicator for the phase transition of lipid membrane and membrane injury [41]. Thus it means that the insertion of the 2NCb with lipid membrane could cause the changes in membrane fluidity.

## Discussion

It has been widely accepted that Aβ oligomers rather than large fibrils exert cytotoxicity to neurons by disrupting the membrane integrity and increasing the membrane conductance. Moreover, Aβ dimer is the smallest aggregate and toxic unit among oligomers of different sizes. Therefore, understanding its structural and dynamics properties is quite useful to prevent the formation and toxicity of the Aβ42 oligomers. In this study, we perform molecular dynamic simulations on four Aβ dimers with different arrangements of Aβ peptides in the lipid bilayers. This study aims to investigate the impact of the aggregate pattern of two Aβ peptides forming the dimer on the structural stability of the dimer and the disruption to the biological membrane.

On the basis of MD results, we observe that all four Aβ dimers are stably embedded into the membrane, and their embedded positions within the membrane do not considerably change during the simulation. However, because the relative position of two Aβ42 peptides is greatly different particularly at the N-terminus, the distance between the N-terminus of two Aβ42 peptides varies among the four dimers. Besides the 2NCb, this distance is increased leading to no formation of the inter-peptide hydrogen bonds at the N-terminus for the three Aβ42 dimers. Without the interaction of inter-peptide hydrogen bonds, the β-strand conformations at the N-terminus can not be stably maintained and easily convert to turn or random coil structure. This structural transformation may further impair the stability of Aβ42 dimer and its aggregated ability to form the larger oligomers. According to this assumption, the 2NCb is considered to be the most stable conformation among the four Aβ42 dimers. Consequently it can serve as a primary aggregate unit of Aβ42 oligomers.

In addition, we further compare the effect of the four Aβ42 dimers on the membrane. The results demonstrate that the insertion of Aβ42 dimer into the membrane can considerably decrease the average APL of the membrane. But this decrease effect is largely dependent on the distance to the location of Aβ42 dimer within the membrane. Moreover, this effect is also related to the secondary structure forms. The β-strand structure causes the larger decrease of APL, whereas the transformation of the β-strand into the turn or random coil structure can alleviate the decrease of APL.

## Conclusions

Altogether, we conclude that the aggregate pattern of two Aβ peptides forming the dimer imposes an influence on the stability of the Aβ42 dimer within the membrane and the aggregate ability to form the larger oligomer. Furthermore, the Aβ dimers with different arrangement of peptides also exhibit the damage effect of different extents on the membrane. Based on the results, the 2NCb is considered as a possible structural unit and potentially toxic form among the four investigated Aβ42 dimers. This result is useful for better understanding the initial aggregation of Aβ42 oligomers. Moreover, this study also provides an insight into how to effectively prevent the aggregation of Aβ oligomers and reduce their damage on the cell membrane.

## Supporting information

S1 Fig Comparison of the mass density profiles for water (black), head-group of DPPC (red) and three Aβ42 dimers, CNNC, NCNC and NCCN, during the fist and last 100 ns of simulation. (TIF)

S2 Fig Comparsion of the final snapshot of four Aβ42 dimers from MD simulation. The Aβ42 peptide is shown in carton. The polar and apolar regions of Aβ42 peptide are colored in blue and red, respectively. The water molecules and phosphorus atoms are shown as Van der Waals spheres of different colors and sizes. The water molecules on the surface of DPPC bilayer are colored in light blue, and the phosphorus atoms within the upper and lower layers are colored in yellow and orange. The hydrophobic core (acyl chains) in DPPC is displayed as line in cyan.
(TIF)

S3 Fig RMSD of all backbone atoms in the four Aβ42 dimers within the DPPC membrabe throughout the 400 ns simulation. The RMSD values are plotted with respect to the structure after equilibration.
(TIF)

S4 Fig RMSF values for all the backbone atoms of four Aβ42 dimers. The 400 ns MD simulation was used in the calculations. The N-terminal polar and C-terminal apolar regions of Aβ42 peptide are shown in blue and gray bar under X-axis, respectively.
(TIF)

S5 Fig Comparison of average secondary structure content, β-strand, turn and coil, for four aβ42 dimers. (TIF)

S6 Fig Comparison of APL for both the top and bottom leaflets between the DPPC membranes inserted with four Aβ42 dimers during the extended 150 ns MD simulation. (TIF)

S7 Fig Average S_CD_ as function of the carbon atom along the lipid tails for the membranes inserted with four Aβ42 dimers, pure DPPC membrane and the experimental values. (TIF)

S8 Fig Radical distribution functions (RDF) for DPPC molecules with respect to Aβ42 dimers. (TIF)
