# Supplementary figures and images for: Study of structural stability and damaging effect on membrane for four Aβ42 dimers
Source: PLoS One. 2017 Jun 8;12(6):e0179147. doi: 10.1371/journal.pone.0179147 (PMC5464659; doi:10.1371/journal.pone.0179147)

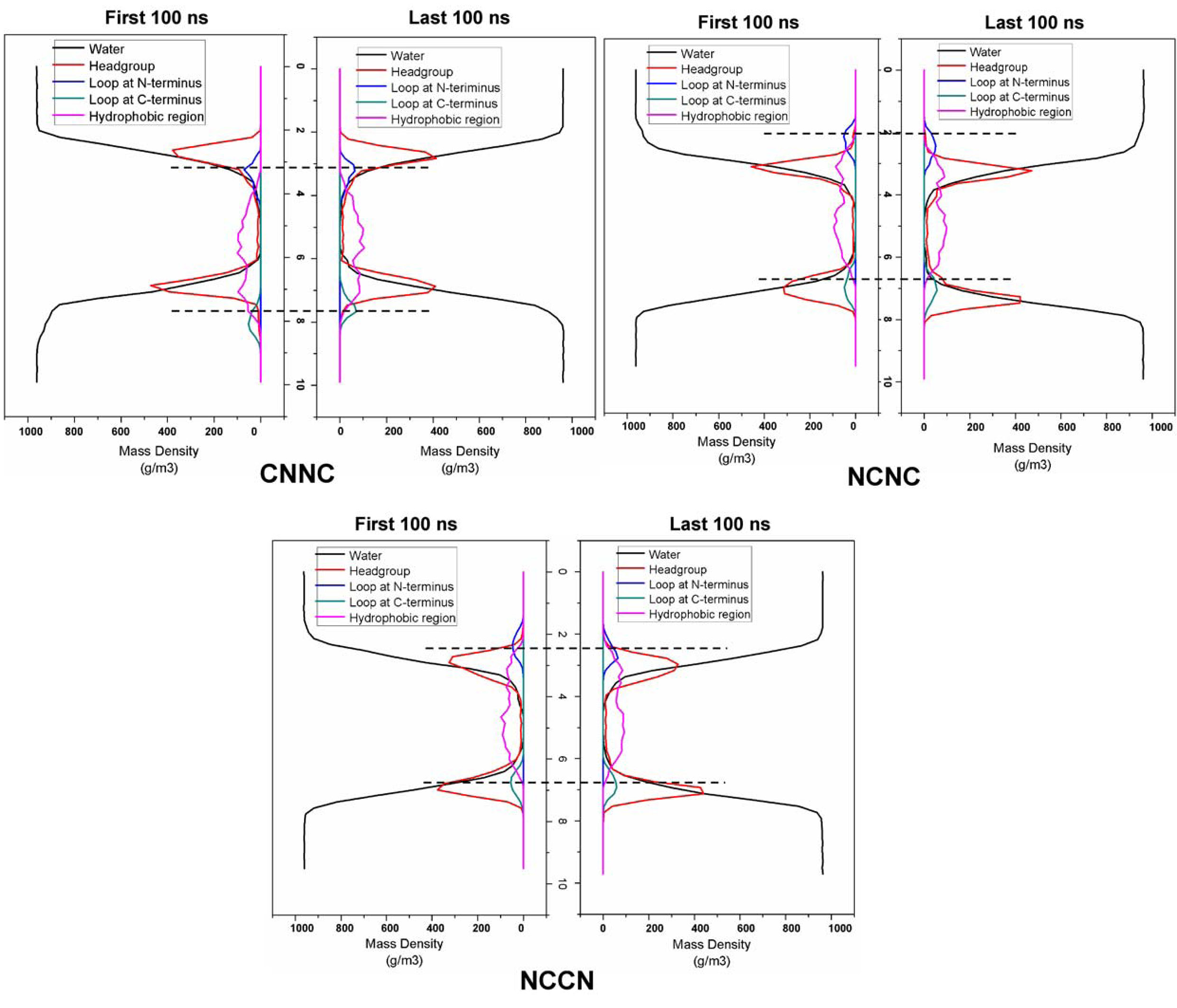

Supplement: S1 Fig — (TIF) [file pone.0179147.s001.tif]

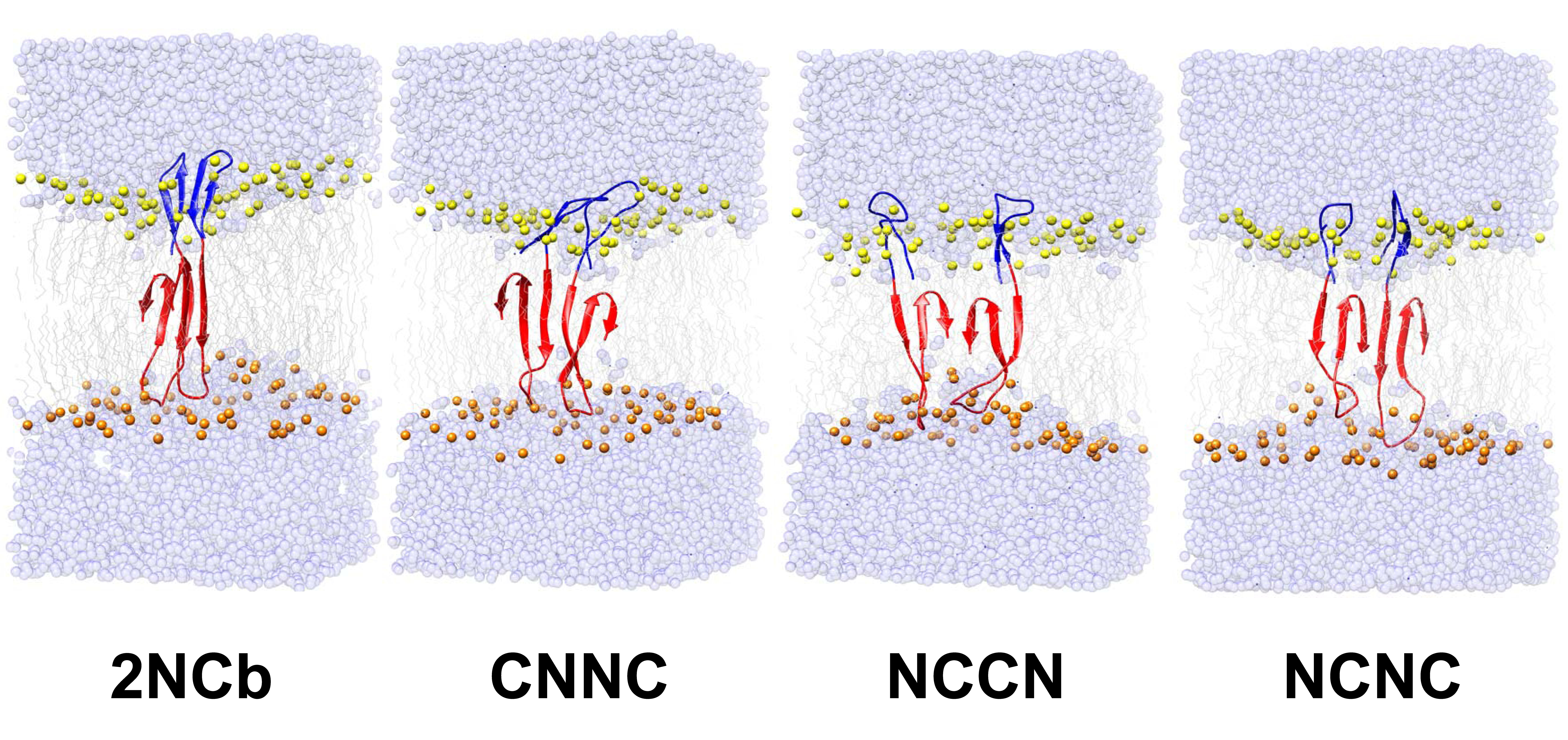

Supplement: S2 Fig — The Aβ42 peptide is shown in carton. The polar and apolar regions of Aβ42 peptide are colored in blue and red, respectively. The water molecules and phosphorus atoms are shown as Van der Waals spheres of different colors and sizes. The water molecules on the surface of DPPC bilayer are colored in light blue, and the phosphorus atoms within the upper and lower layers are colored in yellow and orange. The hydrophobic core (acyl chains) in DPPC is displayed as line in cyan. (TIF) [file pone.0179147.s002.tif]

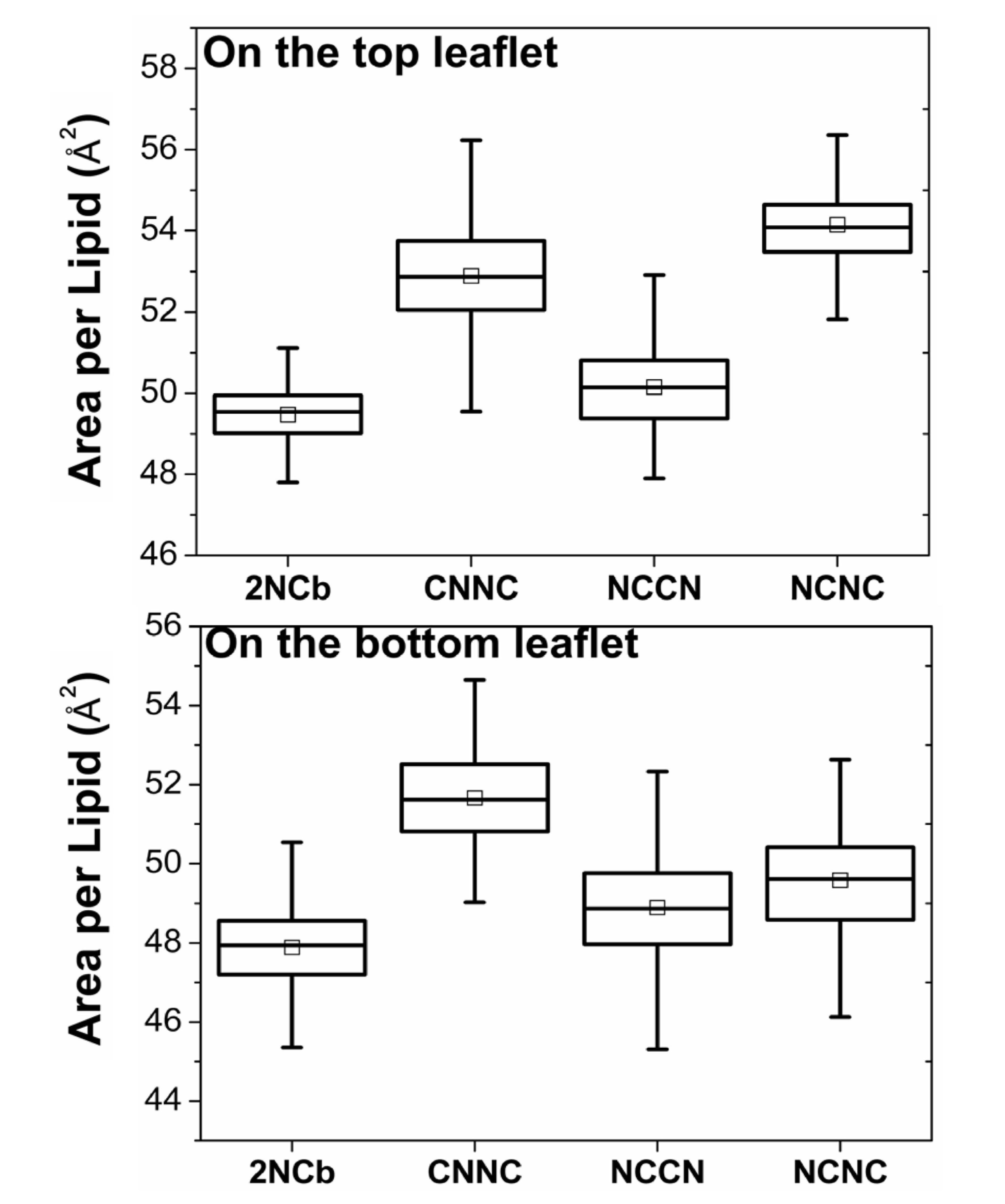

Supplement: S6 Fig — (TIF) [file pone.0179147.s006.tif]

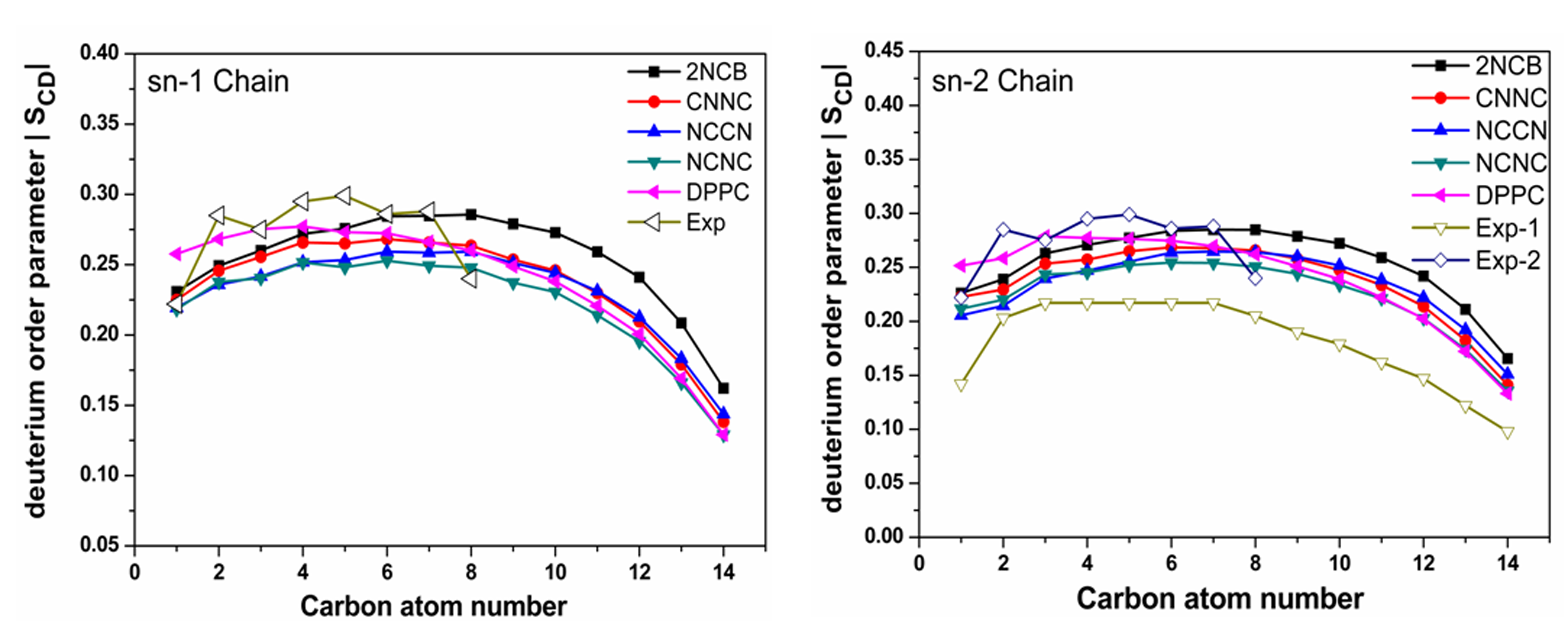

Supplement: S7 Fig — (TIF) [file pone.0179147.s007.tif]
